# Activation of β-catenin signaling in aggrecan-expressing cells in temporomandibular joint causes osteoarthritis-like defects

**DOI:** 10.1038/s41368-018-0016-z

**Published:** 2018-04-23

**Authors:** Tianqian Hui, Yachuan Zhou, Tingyu Wang, Jun Li, Shanxing Zhang, Lifan Liao, Jianhong Gu, Ling Ye, Lan Zhao, Di Chen

**Affiliations:** 1 Department of Pediatric Dentistry, Peking University School and Hospital of Stomatology, Beijing, China; 2 State Key Laboratory of Oral Diseases & National Clinical Research Center for Oral Diseases & West China Hospital of Stomatology, Sichuan University, Chengdu, China; 3 Department of Orthopaedic Surgery, Rush University Medical Center, Chicago, USA; 4 Department of Pharmacy, Shanghai Ninth People’s Hospital, Shanghai JiaoTong University School of Medicine, Shanghai, China; 5 Department of Medical Cell Biology and Genetics, Shenzhen Key Laboratory and the Center for Anti-Ageing and Regenerative Medicine, Shenzhen University Medical School, Shenzhen, China

## Abstract

β-Catenin plays a critical role in cartilage formation and development. To further understand the role of β-catenin in osteoarthritis (OA) development in temporomandibular joint (TMJ), we have generated *β-catenin* conditional activation mice (*β-cat*(*ex3*)^*Agc1CreER*^) by breeding *Agc1-CreER* mice with *β-catenin*^*flox*(*ex3*)/*+*^ mice. Results of histologic analysis showed the progressive TMJ defects in 3- and 6-month-old *β-cat*(*ex3*)^*Agc1CreER*^ mice (tamoxifen induction was performed at 2 weeks of age), including decreased chondrocyte numbers in the superficial layer associated with less Alcian blue staining, increased numbers of hypertrophic chondrocytes in deep layers, and rough articular surface. Compared to the TMJ phenotype of *β-cat*(*ex3*)^*Col2CreER*^ mice, *β-cat*(*ex3*)^*Agc1CreER*^ mice showed much severe morphological defects in the superficial layer of TMJ. This may reflect that *Agc1-CreER* mice could efficiently target cells in the superficial layer of TMJ. Results of immunostaining showed significantly increased expression of MMP13, Col-X, Adamts4, and Adamts5 in TMJ of *β-cat*(*ex3*)^*Agc1CreER*^ mice. Results of proliferating cell nuclear antigen (PCNA), Ki67, and terminal deoxinucleotidyl transferase-mediated dUTP-fluorescein nick end labeling (TUNEL) staining further demonstrated that cell proliferation was decreased and cell apoptosis was increased in condylar cartilage of *β-cat*(*ex3*)^*Agc1CreER*^ mice. Our findings indicate that abnormal upregulation of β-catenin in TMJ leads to defects assembling to OA-like phenotype, further demonstrating that β-catenin plays a critical role in TMJ pathogenesis.

## Arthritis: Seeking relief for aching jaws

Therapies targeting a cartilage-regulating signaling protein could mitigate painful damage inflicted on the jaw by age-related osteoarthritis. Many older individuals experience degeneration of the temporomandibular joint (TMJ), where the upper and lower jaw connect. Researchers led by Di Chen of Rush University Medical School in Chicago, USA, have developed a genetically-modified mouse model that reveals a likely molecular driver for TMJ osteoarthritis. Previous studies have implicated a protein called β-catenin in this process, and Chen’s team generated mice in which β-catenin levels can be selectively boosted in cartilage-forming cells at skeletal joints. This increased β-catenin markedly altered the organization of TMJ cartilage, with decreased cell proliferation and increased cell death. The effects were strikingly similar to human osteoarthritis, and the researchers hypothesize that compounds that counter β-catenin could offer useful treatments for this condition.

## Introduction

The temporomandibular joint (TMJ) is one of the most common sites affected by osteoarthritis (OA). It has been reported that up to 10 million Americans suffer from TMJ disorders (TMDs) each year and 14.56% of mainland Chinese patients with TMD had radiographic signs of OA.^1,2^ Among TMDs, OA is the most prevalent degenerative disease.^3^ TMJ OA is characterized by cartilage degradation, alterations of subchondral bone remodeling, chronic pain, and joint dysfunction.^4,5^ Although TMJ OA is a common degenerative joint disease that affects TMJ cartilage during the aging process, the pathological mechanisms of this disease remain largely unknown.^6^

Canonical Wnt/β-catenin signaling plays an important role in the development and progression in multiple forms of arthritis, such as OA,^7^ spondyloarthritis,^8–10^ and diffuse idiopathic skeletal hyperostosis.^11–13^ It has been shown that conditional activation of β-catenin in knee joint cartilage and intervertebral disc cartilage leads to knee OA and disc tissue degeneration.^7,14^ In most recent studies, we also found that activation of β-catenin signaling in facet joint also causes severe OA-like phenotype (unpublished data). Our goal is to have comprehensive understanding of the role of Wnt/β-catenin signaling in the pathogenesis of arthritis.

TMJ OA is one of the important forms of OA and is a common dental disease. The pathological progression of TMJ OA is considered to be a similar disease as knee OA.^15^ In previous studies, we generated *β-cat*(*ex3*)^*Col2CreER*^ mouse model and demonstrated that dysregulation of β-catenin causes OA-like cartilage degeneration in the TMJ tissue.^16^ We suggest that β*-*catenin is a critical molecule in OA pathogenesis. Interestingly, there is no significant change in the superficial zone of TMJ in *β-cat*(*ex3*)^*Col2CreER*^ mice. And cell proliferation and apoptosis was not changed upon *β-catenin* activation in this mouse model.^16^ TMJ condylar cartilage is comprised of dense extracellular collagen fibers and proteoglycans.^17^ The condylar cartilage is divided into the superficial, middle, and deep layers.^18^ The superficial and/or middle zones of condylar cartilage have been identified as regions enriched with highly proliferative cells.^19^ Mandibular condylar chondrocyte apoptosis and extracellular matrix degradation play an important role in the development of cartilage degeneration in TMJ OA.^20,21^ Moreover, activation of chondrocyte hypertrophy with low metabolism followed by apoptosis in the condylar cartilage is also considered to be part of the disease pathology associated with condylar cartilage degeneration.^22^ We propose that the *β-cat*(*ex3*)^*Col2CreER*^ mice might not be able to fully reveal the pathogenesis of TMJ OA.

We have recently examined the targeting specificity and recombination efficiency of *Agc1-CreER*^*T2*^ mice in TMJ tissue and found that *Agc1-CreER*^*T2*^ mice could efficiently target entire condylar cartilage, including superficial, middle, and deep layers. We decided to use this mouse model to re-evaluate the functions of β-catenin in TMJ tissue using the new *β-cat*(*ex3*)^*Agc1ER*^ conditional activation mouse model. It has been suggested that mechanisms of the aggrecan- or collagen-induced arthritis are very different.^23^ This may be related to the difference of their expression patterns in the condylar cartilage. Another advantage of using *Agc1-CreER*^*T2*^ mice is that these mice could target cartilage tissue in adult animals.^24^ In the present study, we have used *Agc1-CreER*^*T2*^ mice to drive β-catenin overexpression and determined the pathogenesis caused by β-catenin activation in the TMJ tissue. In our study, we explored whether overexpression of β-catenin in aggrecan*-*expressing chondrocytes could lead to cartilage matrix degradation and affect cell proliferation and apoptosis, which may contribute to the OA phenotype observed in *β-cat*(*ex3*)^*Agc1CreER*^ mice.

## Results

### High Cre-recombination efficiency and *β-catenin* activation in *β-cat*(*ex3*)^*Agc1CreER*^ mice

**Fig. 1:**
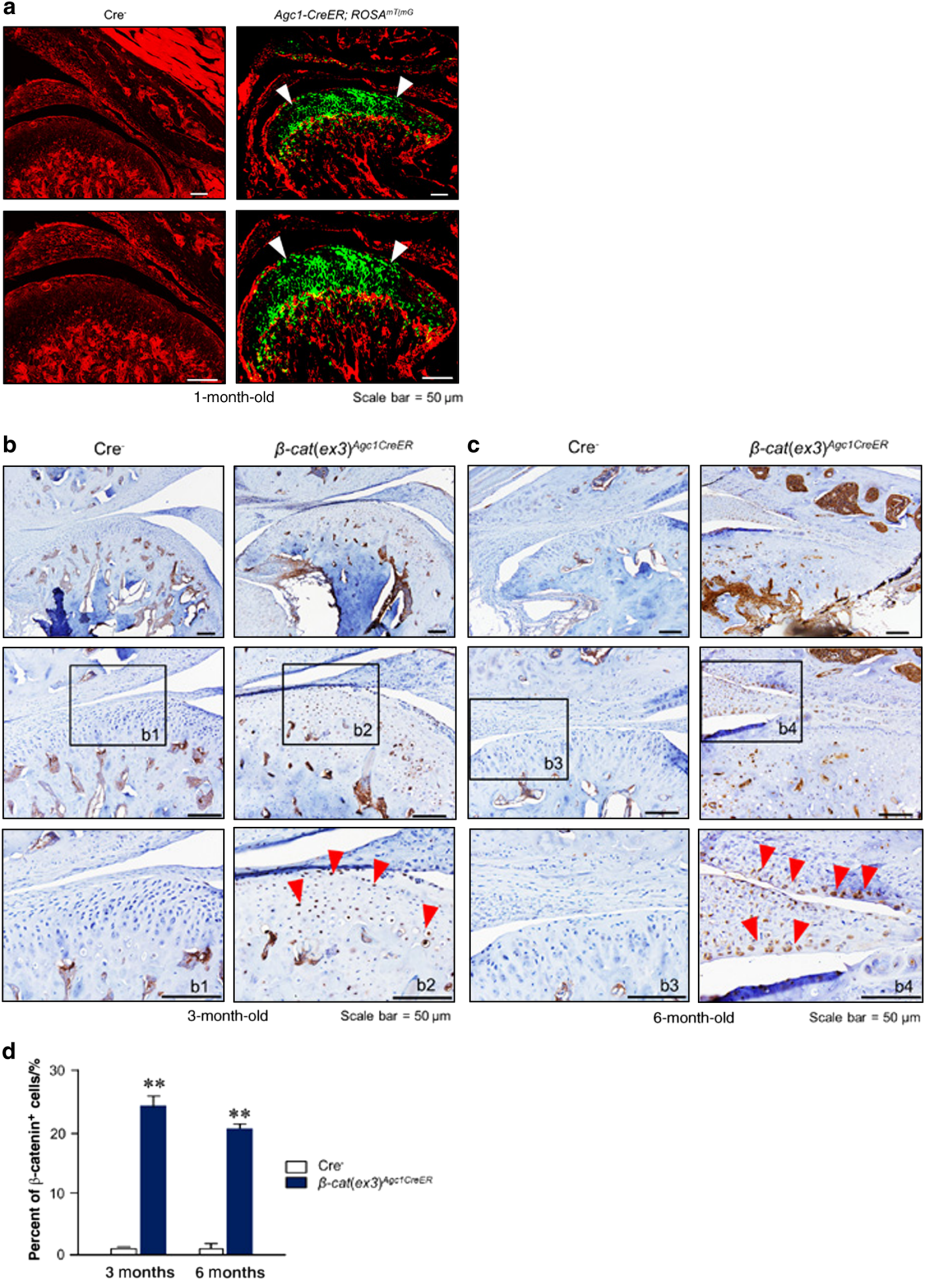
*Agc1-CreER* directed Cre recombination in temporomandibular joint (TMJ) chondrocytes. **a** *Agc1-CreER*^*T2*^; *ROSA*^*mT/mG*^ mice were generated by breeding *Agc1-CreER*^*T2*^ transgenic mice with *ROSA*^*mT/mG*^ reporter mice. TMJ samples were harvested from 1-month-old mice after they were injected with tamoxifen at the age of 2 weeks for 5 consecutive days. Histologic sections from *ROSA*^*mT/mG*^ mice (Cre^−^) and *Agc1-CreER*^*T2*^*; ROSA*^*mT/mG*^ mice were analyzed using fluorescence microscopy. High efficiency of Cre recombination (white arrowheads: *Agc1-CreER*^*T2*^ targeting cells) in the TMJ chondrocytes, including the superficial, middle, and deep layers of condylar chondrocytes was found in *Agc1-CreER*^*T2*^; *ROSA*^*mT/mG*^ mice. **b**, **c** Immunohistochemical (IHC) analysis showed that β-catenin expression was significantly increased in chondrocytes of 3- and 6-month-old *β-cat*(*ex3*)^*Agc1CreER*^ mice. Red arrowheads indicate β-catenin-positive chondrocytes. **d** Quantitative analyses of β-catenin-positive chondrocytes. A significant increase in the numbers of β-catenin-positive cells was observed in *β-cat*(*ex3*)^*Agc1CreER*^ mice compared to Cre^−^ mice (**P < 0.01; values are expressed as mean ± standard erros; *n* = 5 per group)

To evaluate the *Agc1-Cre* expression and recombination efficiency in the TMJ cartilage, *Agc1-CreER*^*T2*^ mice were bred with *ROSA*^*mT/mG*^ reporter mice to generate *Agc1-CreER*^*T2*^*; ROSA*^*mT/mG*^ mice. Tamoxifen was administered when the mice were aged 2 weeks and TMJ samples were harvested at 1 month. The red fluorescent image of condylar cartilage revealed no recombination in Cre-negative control mice (Fig. 1a). The green-labeled chondrocytes in *Agc1-CreER*^*T2*^*; ROSA*^*mT/mG*^ mice showed *Agc1*-expressing cells in the superficial, middle, and deep layers of condylar chondrocytes (Fig. 1a). We then generated *β-cat*(*ex3*)^*Agc1CreER*^ mice by crossing *Agc1-CreER*^*T2*^ mice with *β-catenin*(*ex3*)^*flox/flox*^ mice. Tamoxifen was administered to 2-week-old mice and condylar cartilage samples were harvested from these mice at 3 and 6 months of age. Immunohistochemical (IHC) results showed that *β-catenin* was overexpressed in the majority of condylar chondrocytes at 3- and 6-month-old mice (Fig. 1b, c). There were few β-catenin-positive cells in the chondrocytes of Cre^−^ mice. However, in *β-cat*(*ex3*)^*Agc1CreER*^ mice, *β-catenin* expressed in the superficial, middle, and deep layers of condylar chondrocytes indicating that β-catenin in the chondrocytes was significantly increased compared to the Cre^−^ mice (Fig. 1b, c). The numbers of *β-catenin*-positive cells in TMJ cartilage were significantly higher in *β-cat*(*ex3*)^*Agc1CreER*^ mice compared to Cre^−^ mice (Fig. 1d). These results demonstrated that *Agc1-CreER*^*T2*^ mice could target the chondrocytes of TMJ with high efficiency and drive β-catenin activation in condylar chondrocytes.

### Conditional activation of *β-catenin* induced condylar cartilage defects

**Fig. 2:**
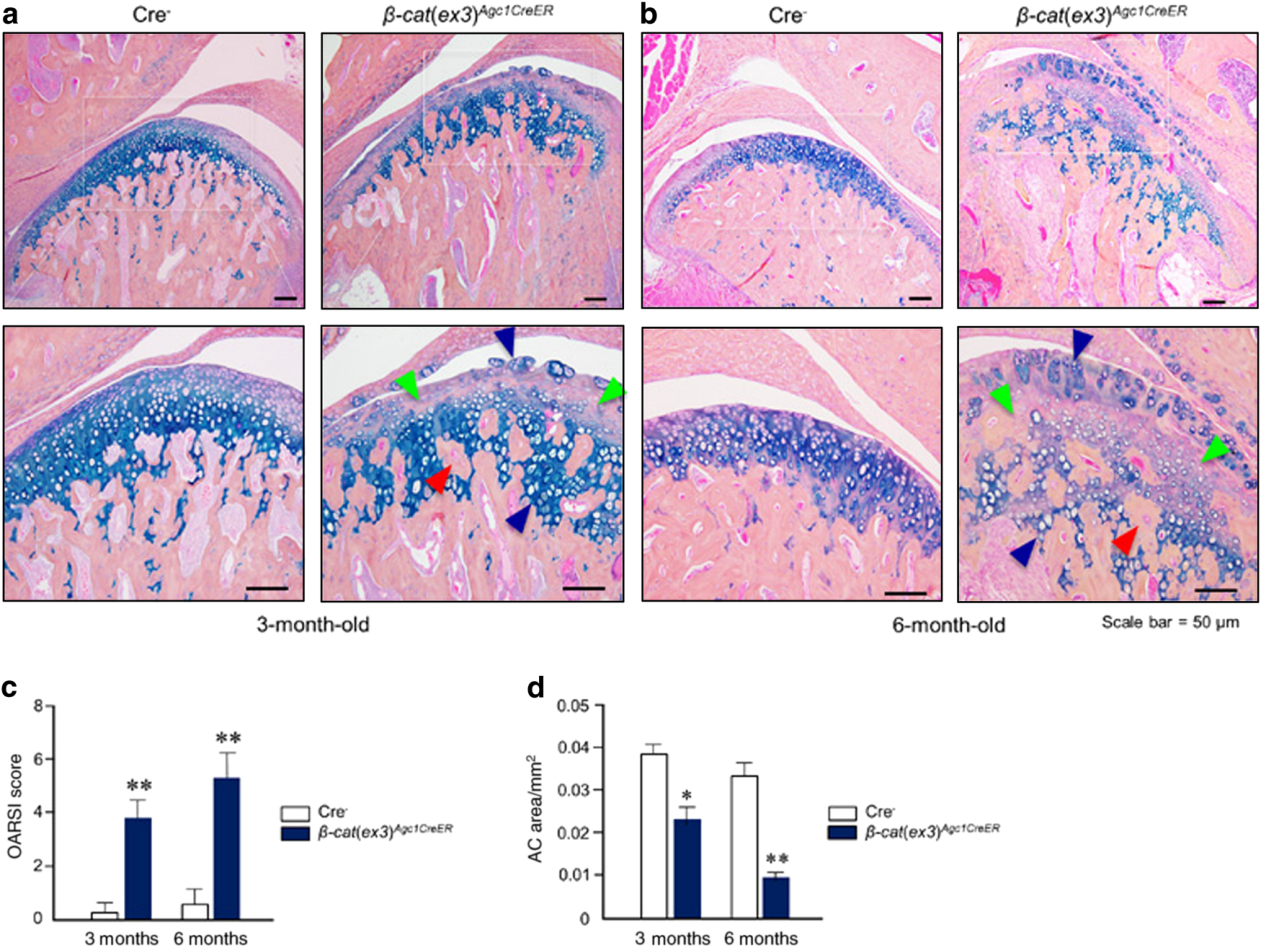
*β-Catenin* conditional activation mice (*β-cat*(*ex3*)^*Agc1CreER*^) show a progressive osteoarthritis (OA)-like phenotype in temporomandibular joint (TMJ) tissue. **a** TMJ samples were dissected from 3- and 6-month-old mice and Alcian blue/hematoxylin staining was performed. *β-cat*(*ex3*)^*Agc1CreER*^ mice displayed early signs of an OA-like phenotype, including increased numbers of hypertrophic chondrocytes, the rough articular surface (blue arrowheads), loss of articular chondrocytes (green arrowheads), and new woven bone formation inside hypertrophic chondrocyte areas (red arrowheads). **b** Loss of cartilage tissue (green arrowheads), increased numbers of hypertrophic chondrocytes and rough articular surface (blue arrowhead), and new woven bone formation inside the hypertrophic chondrocyte areas (red arrowhead) were observed in 6-month-old *β-cat*(*ex3*)^*Agc1CreER*^ mice. **c** Analysis using the scoring system recommended by the Osteoarthritis Research Society International (OARSI) revealed cartilage destruction in 3- and 6-month-old *β-cat*(*ex3*)^*Agc1CreER*^ mice (**P < 0.01, versus Cre^−^ mice; *n* = 5 per group). **d** Histomorphometric analysis showed that TMJ cartilage areas were significantly reduced in 3- and 6-month-old *β-cat*(*ex3*)^*Agc1CreER*^ mice (*P < 0.05, **P < 0.01, versus Cre^−^ mice; values are expressed as mean ± standard errors; *n* = 5 per group)

The role of β-catenin in condylar cartilage was investigated in *β-cat*(*ex3*)^*Agc1CreER*^ mice. Tamoxifen was administered to 2-week-old mice and TMJ samples were harvested from these mice at 3 and 6 months of age. The chondrocytes in the control mice were well organized in the three layers: small and round cells in the top superficial layer; medium-sized cells were present in large numbers in the middle layer; and fewer, bigger, hypertrophic, mature cells in the deep layer (Fig. 2a, b, left panels). In contrast, 3-month-old *β-cat*(*ex3*)^*Agc1CreER*^ mice presented early signs of TMJ OA: decreased chondrocyte numbers in the superficial and middle layer accompanied with less Alcian blue staining in these areas, rough articular surface with numerous rounded chondrocytes often appearing as doublets, and cells in the middle and deep layers illustrated increased numbers of hypertrophic cells. In addition to decreased cellularity of the middle layers of cartilage, clustering of hypertrophic chondrocytes appeared more frequently in the deeper layer; cartilage area scattering and subchondral bone sclerosis were also observed in the *β-cat*(*ex3*)^*Agc1CreER*^ mice compared with age-matched control group (Fig. 2a, right panel). At 6 months of age, increased severity of defects, such as clustering chondrocytes in the superficial and deeper layer, the increasing numbers of hypertrophic chondrocytes, and subchondral new bone formation in condylar cartilage were observed in *β-cat*(*ex3*)^*Agc1CreER*^ mice (Fig. 2b, right panel). We also analyzed the histology sections using the scoring system recommended by the Osteoarthritis Research Society International (OARSI) as previously described.^27,29^ We found that *β-cat*(*ex3*)^*Agc1CreER*^ mice had significantly higher scores for OA damage compared to Cre^−^ mice (Fig. 2c). The histomorphometric analysis also showed significant reductions in articular cartilage area in 3- and 6-month-old *β-cat*(*ex3*)^*Agc1CreER*^ mice (Fig. 2d).

### Changes in the expression of genes encoding for matrix-degradation enzymes in *β-cat*(*ex3*)^*Agc1ER*^ mice

**Fig. 3:**
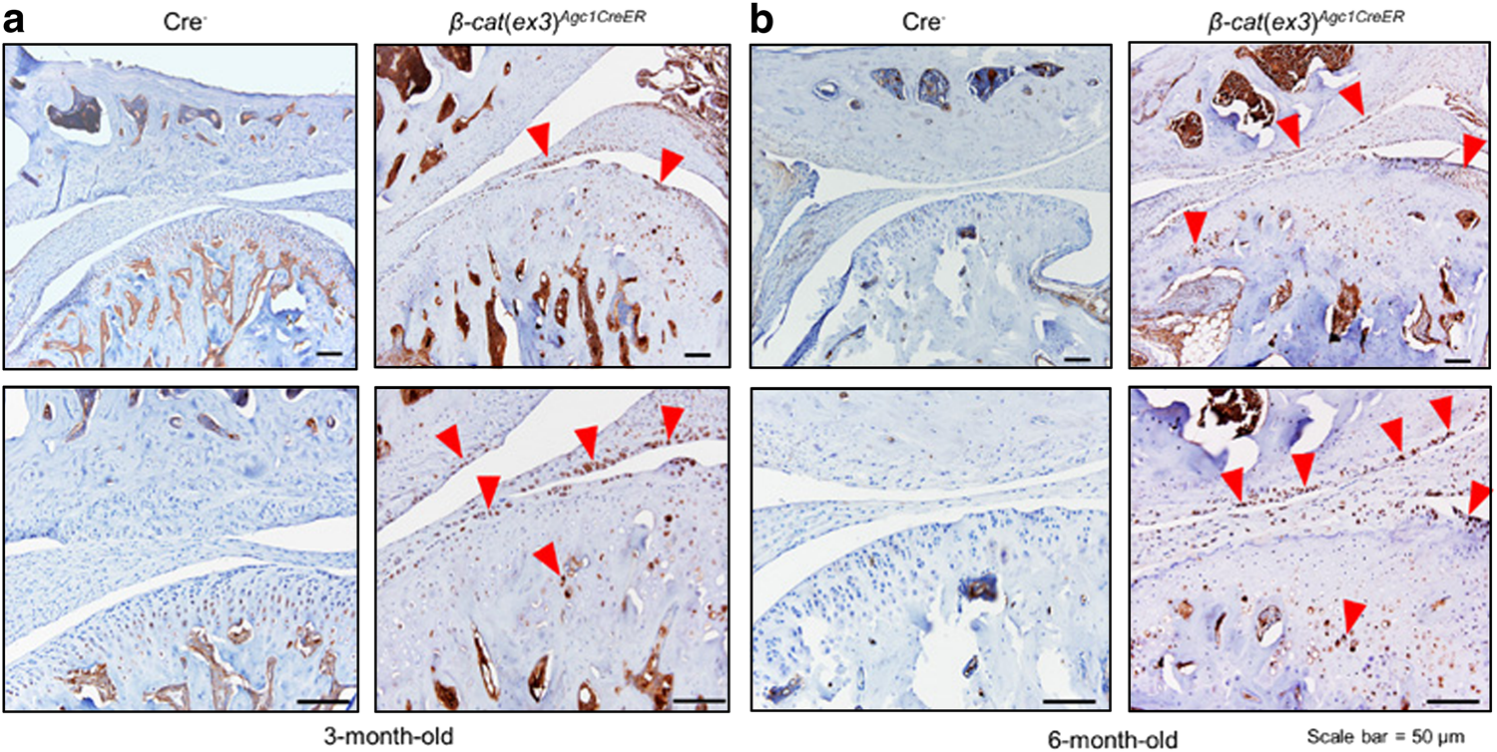
Immunohistochemical (IHC) analysis of MMP13 expression. **a**, **b** MMP13 protein levels were increased in 3- and 6-month-old *β-cat*(*ex3*)^*Agc1CreER*^ conditional activation mice, especially in the superficial zone (red arrowheads: MMP13-positive cells)

**Fig. 4:**
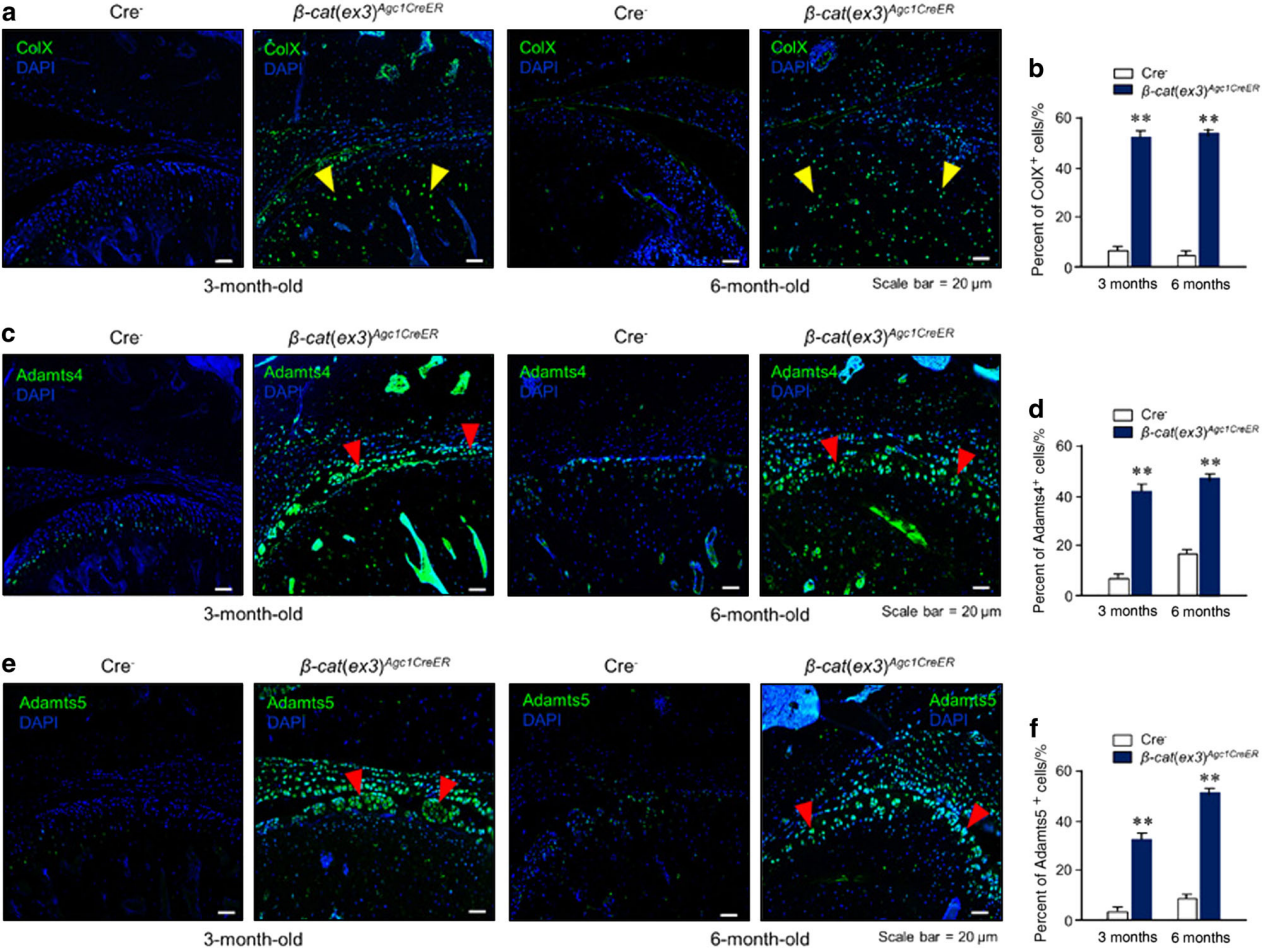
Abnormal expression of the hypertrophic chondrocyte marker and matrix-degradation enzymes in 3- and 6-month-old *β-cat*(*ex3*)^*Agc1CreER*^ conditional activation mice. Immunofluorescence (IF) assays were performed to detect the expression of ColX, Adamts4, and Adamts5 proteins in *β-cat*(*ex3*)^*Agc1CreER*^ mice. **a, b **IF results showed that Co1X expression was significantly increased in the areas that hypertrophic chondrocytes are located in *β-catenin* conditional activation mice . Yellow arrowheads: ColX-positive cells. **c–f **The expression of Adamts4 and Adamts5 was also significantly increased, especially on the surface of TMJ cartilage in *β-cat*(*ex3*)^*Agc1CreER*^ mice (**P < 0.01; values are expressed as mean ± SE; *n* = 5 per group). Red arrowheads: Adamts4- and Adamts5-positive cells

We have previously observed significant upregulation of *Mmp13* and *Adamts5* expression in *β-cat*(*ex3*)^*Col2CreER*^ mice and demonstrated that both matrix metalloproteinase 13 (MMP13) and Adamts5 play important roles in the TMJ OA development in these mice.^16^ We propose that *Mmp13* and *Adamts5* might be the key downstream target genes of β-catenin during TMJ OA development. To further test this hypothesis, we performed IHC and immunofluorescence (IF) assays to determine changes in the expression of these collagenase and aggrecanases. Results of IHC revealed increased MMP13 expression in the *β-cat*(*ex3*)^*Agc1CreER*^ mice, especially in the superficial layer and deeper layer of the condylar cartilage in 3- and 6- month-old mice compared to controls (Fig. 3a, b). In addition, IF results showed significant increased ColX expression in chondrocytes of entire condylar cartilage in *β-cat*(*ex3*)^*Agc1CreER*^ mice compared to controls, indicating that the chondrocytes underwent hypertrophy at this stage (Fig. 4a, b). Furthermore, the expression of cartilage-degrading enzymes, such as Adamts4, and Adamts5 was also increased, especially in the superficial layer of TMJ chondrocytes in *β-cat*(*ex3*)^*Agc1CreER*^ mice (Fig. 4c–f). These results suggest that the activation of β-catenin signaling could lead to chondrocyte hypertrophy and degenerative defects.

### Alterations of cell proliferation and apoptosis in *β-cat*(*ex3*)^*Agc1CreER*^ mice

**Fig. 5:**
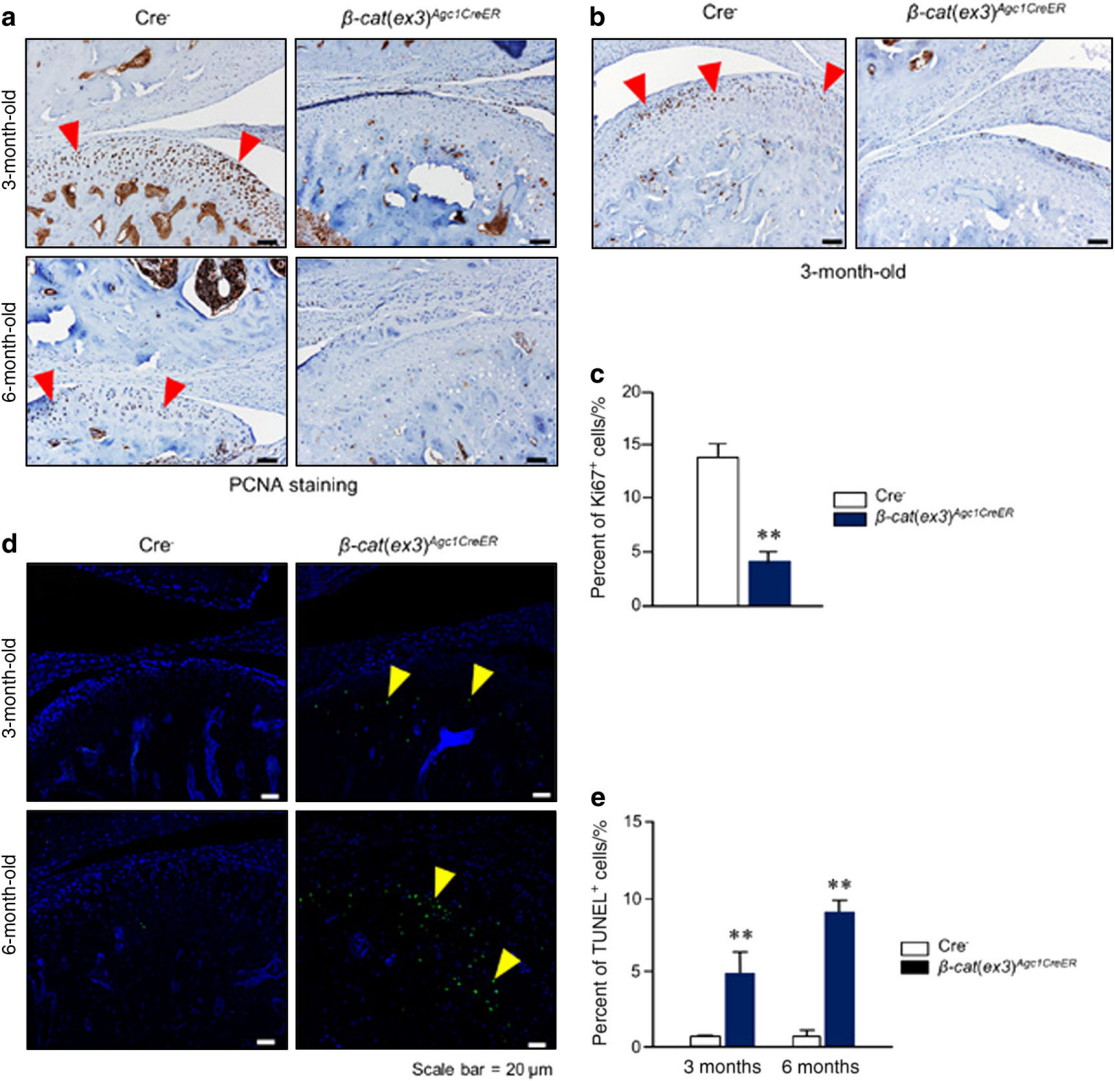
Alteration of cell proliferation and apoptosis in 3- and 6-month-old *β-cat*(*ex3*)^*Agc1CreER*^ conditional activation mice. **a** Results of proliferating cell nuclear antigen (PCNA) staining revealed that cell proliferation was significantly reduced in temporomandibular joint (TMJ) chondrocytes in *β-cat*(*ex3*)^*Agc1CreER*^ mice. Red arrowheads: PCNA-positive cells. **b**, **c** Results of Ki67 staining revealed that cell proliferation, mostly in the middle layer of condylar chondrocytes, significantly reduced in TMJ cartilage in 3-month-old *β-cat*(*ex3*)^*Agc1CreER*^ mice. Red arrowheads: Ki67-positive cells. **d**, **e** Results of terminal deoxinucleotidyl transferasemediated dUTP-fluorescein nick end labeling (TUNEL) staining demonstrated increased numbers of apoptotic cells were detected in TMJ cartilage of *β-cat*(*ex3*)^*Agc1CreER*^ mice (**P < 0.01 versus Cre^−^ mice; values are expressed as mean ± standard errors; *n* = 5 per group). Yellow arrowheads: TUNEL positive cells

To further investigate the pathological process in *β- cat*(*ex3*)^*Agc1CreER*^ mice, proliferating cell nuclear antigen (PCNA), Ki67, and terminal deoxinucleotidyl transferase-mediated dUTP-fluorescein nick end labeling (TUNEL) staining was performed to assess changes in chondrocyte proliferation and apoptosis. In control mice, especially at the 3-month-old, results of PCNA staining showed that abundant proliferating cells were present in the entire TMJ cartilage. However, the PCNA-positive cells were dramatically reduced in the condylar cartilage of 3- and 6-month-old *β-cat*(*ex3*)^*Agc1CreER*^ mice (Fig. 5a). To further analyze changes in cell proliferation, we also performed Ki67 staining and found that numbers of Ki67-positive cells in the middle zones of condylar cartilage were significantly reduced in 3-month-old *β-cat*(*ex3*)^*Agc1CreER*^ mice (Fig. 5b, c). These results suggest that overexpression of *β-catenin* in aggrecan-expressing condylar chondrocytes significantly affects cell proliferation. Data of TUNEL staining demonstrated the increased apoptotic cells, mostly in the deeper layers of the condylar cartilage of the mutant mice compared to that in control mice (Fig. 5d, e). In the control group, only few scattered apoptotic cells were detected in the deeper layer of the condylar cartilage in the mice at 6 months of age. Taking together, these results indicate that conditional activation of *β-catenin* in the TMJ tissue induced degenerative defects that might be partly due to changes in cell proliferation and apoptosis.

## Discussion

In this study, we generated *β-cat*(*ex3*)^*Agc1CreER*^ mouse model and demonstrated that overexpression of *β-catenin* in aggrecan-expressing chondrocytes leads to degenerative defects resembling an OA-like phenotype in condylar cartilage. The TMJ OA is a degenerative disease with age-related joint disorder.^25^ Meanwhile, TMJ disorders mostly affect young women according to recent researches.^26,27^ The *Agc1-CreER*^*T2*^ transgenic mouse model is a valuable tool to investigate the postnatal OA development, allowing chondrocyte-specific gene targeting in an inducible manner.^24^ To determine the role of β-catenin in TMJ OA development in postnatal mice, we decided to induce β-catenin expression in 2-week-old mice. β-Catenin was activated specifically in mature chondrocytes. *β-cat*(*ex3*)^*Agc1CreER*^ mice exhibited TMJ phenotype similar to that of human TMJ OA, including increased chondrocyte hypertrophy observed in the superficial zone of the condylar cartilage, severe loss of articular cartilage at the margins of cartilage tissue, and subchondral sclerosis. In *β-catenin* conditional activation mice, the accelerated catabolic effects (matrix degradation and hypertrophy) may contribute to the eventual loss of the condylar cartilage in this mouse model.

In previous studies, we demonstrated that *β-cat*(*ex3*) ^*Co12CreER*^ mice also showed TMJ OA-like phonotype.^16^ Compared to middle and deep layers, the superficial area of the condylar cartilage is relatively normal. This is probably because the *Col2* gene is not expressed in cells of the superficial layer. *Col2* is mainly expressed in the middle and deep layers,^28^ so the condylar cartilage was not the most efficiently targeted by *Co12-CreER*^*T2*^ mice.^3,29,30^ In the present study, we specifically determine the role of β-catenin signaling in *Agc1-CreER*^*T2*^ targeting cells. Interestingly, we observed that activation of β-catenin signaling in aggrecan-expressing cells leads to dramatic damage in the superficial zone of condylar cartilage. This finding indicates that the superficial zone of the condylar cartilage could be more efficiently targeted by *Agc1-CreER*^*T2*^ mice. Our findings using both transgenic mice clearly demonstrated that the proper level of β-catenin activity is critical for maintaining the integrity of the condylar cartilage in TMJ.

In this study, we also observed significant increases in the expression of collagenase (MMP13) and aggrecanases (Adamts4 and Adamts5) in *β-cat*(*ex3*)^*Agc1CreER*^ mice. In addition, there is a significant increase in the expression of ColX, the most specific marker of hypertrophic chondrocytes, in *β-cat*(*ex3*)^*Agc1CreER*^ mice. Consistent with this, increased numbers of hypertrophic chondrocytes were observed in the condylar cartilage in *β-cat*(*ex3*)^*Agc1CreER*^ mice. MMP13 and Adamts5 are the primary enzymes leading to cartilage degradation.^31,32^ β-Catenin may serve as an important regulator of MMP13 and Adamts5 in hypertrophic chondrocytes. In our study, we used two different β-catenin activation mouse models to demonstrate that proper levels of β-catenin are critical in maintaining condylar cartilage integrity; however, it remains unknown how β-catenin signaling is upregulated during the development of TMJ OA.

Previous reports revealed that cell proliferation and apoptosis in condylar cartilage could also be involved in OA development.^20,33^ The rat TMJ OA model showed histological changes, including reduced chondrocytes proliferation and increased chondrocytes apoptosis.^34^ It has also been reported that the OA is caused by excessive chondrocyte apoptosis.^35^ The superficial and/or middle zones of normal condylar cartilage have been identified as regions enriched in cells that are highly proliferative.^19^ In the present study, in addition to the cartilage degradation we also demonstrated decreased chondrocyte proliferation and increased chondrocyte apoptosis in *β-cat*(*ex3*)^*Agc1CreER*^ mice. These changes could also contribute to the development of TMJ OA. The increased numbers of TUNEL-positive chondrocytes may reflect the enhancement of chondrocyte differentiation in the middle zone of the condylar cartilage in *β-cat*(*ex3*)^*Agc1CreER*^ mice.^36^ This is consistent with the notion that cell apoptosis of mandibular condylar could be responsible for the development and progression of TMJ OA.^20,34^

In summary, our study revealed that, in addition to changes in the middle and deep zones, the morphology and function of the superficial zone of cartilage could also be regulated by β-catenin signaling. We suggest that β-catenin may play important roles in chondrocyte proliferation, differentiation, and apoptosis in the condylar cartilage. Dysregulation of β-catenin signaling in chondrocytes of condylar cartilage may cause significant changes in chondrocyte function, leading to TMJ OA development. TMJ β-catenin signaling may be served as a potential therapeutic target for the development of drugs to treat TMJ OA.

## Materials and methods

### Animals

*Agc1-CreER*^*T2*^ transgenic mice^24^ and *ROSA*^*mT/mG*^ (membrane-Tomato/membrane-Green) reporter mice^37^ were obtained from Jackson Laboratories (Bar Harbor, ME, USA). *β-catenin*(*ex3*)^*flox/flox*^ mice were originally reported by Harada et al.,^38^ and we have used these mice in our previous studies.^7,14,16^ *β-cat*(*ex3*)^*Agc1CreER*^ mice and the Cre-negative littermates were generated. Tamoxifen (Sigma, St. Louis, MO, USA) was administered into 2-week-old mice by intraperitoneal (i.p.) injection (1 mg per 10 g body weight for 5 consecutive days), *n* = 5 in each group. The animal protocol of this study has been approved by the IACUC of the Rush University and all experimental methods and procedures were carried out in accordance with the approved guidelines.

### Cre-recombination efficiency

*ROSA*^*mT/mG*^ mice contain two *loxP* sites on either side of the mT cassette. Mice express red fluorescence in all cell types and tissues before Cre-recombination and green fluorescence signal can be detected after Cre-recombination.^37^ *Agc1-CreER*^*T2*^ mice were bred with *ROSA*^*mT/mG*^ mice to generate *Agc1-CreER*^*T2*^*; ROSA*^*mT/mG*^ mice. Tamoxifen was administered into 2-week-old mice by i.p. injection (1 mg per 10 g body weight for 5 days). Skulls were dissected after the mice were sacrificed at age 1 month, fixed in 0.2% glutaraldehyde at 4 °C for 2 days, followed by washing three times with phosphate buffered saline (PBS). Samples were decalcified in 14% EDTA for 3 weeks, cryo-protected in 30% sucrose at 4 °C for 3 days and then embedded and processed for frozen sections. Three-μm-thick sections were imaged with a fluorescence microscope.

### Histology and histomorphometry

We dissected the skulls from *β-cat*(*ex3*)^*Agc1CreER*^ mice and their corresponding Cre-negative control mice. Samples were fixed in 10% neutral buffered formalin (VWR, Radnor, PA, USA) for 3 days, then decalcified with formic acid (Decal Chemical Corp., Suffern, NY, USA) for 14 days. Samples were processed and embedded in paraffin. Three-μm-thick mid-sagittal sections at three different levels (50 μm apart) were cut from the medial compartment of the TMJ. The sections were stained with Alcian blue/hematoxylin and eosin for morphologic analysis. Three slides per mouse, five mice per group, were analyzed in the experiment. The histology data were further analyzed with OARSI scoring system as previously described.^39,40^ We also quantified the cartilage area using the OsteoMeasure software (OsteoMetrics, Inc., Atlanta, GA, USA).

### IHC and IF

The paraffin sections were baked at 65 °C overnight. Slides were then deparaffinized and rehydrated. Dako endogenous blocking reagent (S2003, Dako, Carpinteria, CA, USA) was then used to quench endogenous peroxidase for 15 min. Non-specific binding sites were blocked with 1:10 normal horse/goat serum (S-2000, Vector Laboratories, Burlingame, CA, USA) for 30 min at room temperature. Primary antibodies: 1:400 dilution of MMP13 (ab39012, Abcam, Cambridge, UK), 1:1 000 dilution of ColX (ab49945, Abcam, Cambridge, UK); 1:400 dilution of Admts4 (ABT178, Millipore, Billerica, MA), and 1:500 dilution of Adamts5 (ab41037 Abcam, Cambridge, UK) were added, and the slides were incubated at 4 °C overnight. For IHC assays, the secondary biotinylated goat anti-mouse antibody (BA-9200, Vector Laboratories) at the dilution of 1:200 was added for 30 min on the second day, followed by incubation with 1:250 streptavidin (21130, Pierce, Rockford, IL, USA) for 30 min. Positive staining was detected by Romulin AEC Chromagen (Biocare Medical RAEC810L, Concord, CA, USA). For IF staining, an appropriate secondary antibody conjugated to a fluorescence probe was added, incubated at room temperature for 1 h, rinsed in PBS, and mounted in an anti-fading mounting media (Vector Laboratories, Burlingame, CA). Results were obtained using an Olympus BX43 upright microscope (Olympus Optical, Tokyo, Japan).

### Cell proliferation and apoptosis assays

We dissected TMJ tissues from *β-cat*(*ex3*) ^*Agc1CreER*^ mice and Cre-negative controls. Samples were fixed in 10% formalin, decalcified, and embedded in paraffin. The condyles were sectioned into serial sections at 3-μm-thick in an anterior–posterior direction. Cell proliferation was carried out using anti-PCNA and anti-Ki67 antibodies at the dilution of 1:200 (abl8197, ab16667, Abcam, Cambridge, UK) and 1:2 000, respectively, as previously described.^16^ Apoptosis assay was carried out using a TUNEL Assay Kit according to the manufacturer’s instructions (G3250, Promega, Madison, WI, USA).

### Statistical analysis

The values are presented as mean ± standard error. Statistical difference between groups was evaluated using one-way analysis of variance followed by Tukey–Kramer test and Student’s *t*-test with the SPSS13.0 statistical software. *P < 0.05 and **P < 0.01 are considered as significant difference between groups.
